# Das Thierarztliche Unterrichts Wesen Deutschlands

**Published:** 1890-09

**Authors:** 


### REVIEW DEPARTMENT.

Das thierarztliche Unterrichts wesen Deutschlands: Ein gendenkblatt aüs Anlass der Feier des 100 jährigen Bestehens der thierärztlichen Hochschule zü Berlin, bearbeitet von Dr. Georg Schneidenmuhl. Leipzig. Verlag von Arthur Felix, 1890.[1]

This is one of the most interesting and useful memorials of the history of comparative pathology extant. It opens with a narrative of veterinary surgical history in general, continues with a biography of the great Gerlach —who may be aptly styled the Virchow of this branch of science—and concludes with a history of various parliamentary debates and the reforms to which these led.

In dealing with the claims of rival nations great impartiality is shown; in fact, if the German scientific writer errs in any direction in this field, it is usually in the safe if rather undignified one of self-depreciation. We learn that Claude Bourgelat founded the first of all veterinary schools - that at Lyons, while Turin (1769), Gottingen (1771), Vienna (1777), London (1792), Madrid (1793), Wilna (1804), Bern (1806), Lisbon (1830), Brussels (1832), Constantinople (1849) indicate the successive imitation of this precedent by the chief European lands. The Montreal school is dated from 1866, but the New York one is erroneously given as 1875.

There is one feature in German veterinary life revealed in the pages of this book, and for which its author is not to be held responsible, which, to say the least, strikes an American reader as mawkish and morbid. What possible connection can there be between royalistic hyper-loyalty and veterinary or any other science? Listen! "The students of the Veterinary High School of Berlin, assembled at a grand banquet in Kroll's Hall, feel themselves bound to respectfully offer Your Majesty their vows of fidelity and unconditional[1] obedience." This is followed by the ceremony of

[1] The original has it "irrefragible."

"salamander reiben"—the beer glasses being emptied at a signal, returned and drummed on the table, undoubtedly advancing thus the cause of veterinary science and loyalty! Even the great Lydtin, of Carlsruhe, could not, at the same occasion, repress some sentimental twaddle about "Kaiser Wilhelm, the victorious."

To return to the serious and valuable contents of the book. It appears, from the parliamentary debates, that the profession of veterinary medicine in Germany is threatened with overcrowding, the number of students at Berlin having become quadrupled in fifteen years. It is, therefore, not regarded as desirable to found other or to enlarge the existing schools.

[1] The Veterinary Educational System of Germany: A memorial written on the occasion of the celebration of the ceneennial anniversary of the Veterinary School of Berlin etc.

Here in the United States we do not suffer from an overplus of students nor of colleges, yet the initiated feel tempted, instead of adding to or enlarging the latter, to abolish the majority of them. It is a most fortunate thing for our national sensibilities that Dr. Schneidenmühl passes over the subject of our New York schools in silence.

An excellent portrait of Gerlach constitutes the frontispiece, and this forms an inducement to purchase the book, even by those who do not read German.

